# Renal function estimation and Cockcroft–Gault formulas for predicting cardiovascular mortality in population-based, cardiovascular risk, heart failure and post-myocardial infarction cohorts: The Heart ‘OMics’ in AGEing (HOMAGE) and the high-risk myocardial infarction database initiatives

**DOI:** 10.1186/s12916-016-0731-2

**Published:** 2016-11-10

**Authors:** João Pedro Ferreira, Nicolas Girerd, Pierpaolo Pellicori, Kevin Duarte, Sophie Girerd, Marc A. Pfeffer, John J. V. McMurray, Bertram Pitt, Kenneth Dickstein, Lotte Jacobs, Jan A. Staessen, Javed Butler, Roberto Latini, Serge Masson, Alexandre Mebazaa, Hans Peter Brunner-La Rocca, Christian Delles, Stephane Heymans, Naveed Sattar, J. Wouter Jukema, John G. Cleland, Faiez Zannad, Patrick Rossignol, for the Heart ‘OMics’ in AGEing (HOMAGE) initiative and the High-Risk Myocardial Infarction database initiative

**Affiliations:** 1 INSERM, Centre d’Investigations Cliniques Plurithématique 1433, INSERM U1116, Université de Lorraine, CHRU de Nancy, F-CRIN INI-CRCT, Nancy, France; 2 Academic Cardiology Unit, University of Hull, Castle Hill Hospital, Kingston upon Hull, UK; 3 Université de Lorraine, Institut Elie Cartan de Lorraine, UMR 7502,, Vandoeuvre-lès-Nancy, F-54506 France; 4 CNRS, Institut Elie Cartan de Lorraine, UMR 7502,, Vandoeuvre-lès-Nancy, F-54506 France; 5 Team BIGS, INRIA, Villers-lès-Nancy, F-54600 France; 6 Division of Cardiovascular Medicine, Brigham & Women’s Hospital, Harvard Medical School, Boston, Massachusetts 02115 USA; 7 BHF Cardiovascular Research Centre, University of Glasgow, Glasgow, Scotland UK; 8 Department of Medicine, University of Michigan School of Medicine, Ann Arbor, MA USA; 9 ASH Comprehensive Hypertension Center, Division of Endocrinology, Diabetes, and Metabolism, Department of Medicine, The University of Chicago, Chicago, IL USA; 10 Department of Cardiology, University of Bergan, Stavanger University Hospital, Stavanger, Norway; 11 Studies Coordinating Centre, Research Unit Hypertension and Cardiovascular Epidemiology, KU Leuven Department of Cardiovascular Sciences, University of Leuven, Leuven, Belgium; 12 Cardiology Division, Stony Brook University, Stony Brook, NY USA; 13 Laboratory of Cardiovascular Clinical Pharmacology, IRCCS - Istituto di Ricerche Farmacologiche ‘Mario Negri’, Milan, Italy; 14 Hôpital Lariboisière, Université Paris Diderot, Inserm 942, Paris, France; 15 Cardiovascular Research Institute Maastricht, Maastricht University Medical Centre, Maastricht, The Netherlands Department of Cardiology, Maastricht University Medical Centre, Maastricht, The Netherlands; 16 Department of Cardiology, Maastricht University Medical Center, Postbox 5800, 6202 AZ Maastricht, The Netherlands; 17 Department of Cardiology and Einthoven Laboratory for Experimental Vascular Medicine, Leiden University Medical Center, Leiden, The Netherlands; 18 Interuniversity Cardiology Institute of the Netherlands, Utrecht, The Netherlands; 19 National Heart and Lung Institute, Imperial College London (Royal Brompton and Harefield Hospitals) Department of Cardiology, Castle Hill Hospital, University of Hull, Hull, UK; 20 Centre d’Investigations Cliniques-INSERM CHU de Nancy, Institut Lorrain du Cœur et des Vaisseaux Louis Mathieu, 4 Rue du Morvan, 54500 Vandoeuvre lès Nancy, France

**Keywords:** Population based, Cardiovascular risk, Heart failure and post-myocardial infarction cohorts, Renal function, Glomerular filtration rate formulas, Cardiovascular mortality prediction

## Abstract

**Background:**

Renal impairment is a major risk factor for mortality in various populations. Three formulas are frequently used to assess both glomerular filtration rate (eGFR) or creatinine clearance (CrCl) and mortality prediction: body surface area adjusted-Cockcroft–Gault (CG-BSA), Modification of Diet in Renal Disease Study (MDRD4), and the Chronic Kidney Disease Epidemiology Collaboration (CKD-EPI) equation. The CKD-EPI is the most accurate eGFR estimator as compared to a “gold-standard”; however, which of the latter is the best formula to assess prognosis remains to be clarified. This study aimed to compare the prognostic value of these formulas in predicting the risk of cardiovascular mortality (CVM) in population-based, cardiovascular risk, heart failure (HF) and post-myocardial infarction (MI) cohorts.

**Methods:**

Two previously published cohorts of pooled patient data derived from the partners involved in the HOMAGE-consortium and from four clinical trials – CAPRICORN, EPHESUS, OPTIMAAL and VALIANT – the high risk MI initiative, were used. A total of 54,111 patients were included in the present analysis: 2644 from population-based cohorts; 20,895 from cardiovascular risk cohorts; 1801 from heart failure cohorts; and 28,771 from post-myocardial infarction cohorts. Participants were patients enrolled in the respective cohorts and trials. The primary outcome was CVM.

**Results:**

All formulas were strongly and independently associated with CVM. Lower eGFR/CrCl was associated with increasing CVM rates for values below 60 mL/min/m^2^. Categorical renal function stages diverged in a more pronounced manner with the CG-BSA formula in all populations (higher χ^2^ values), with lower stages showing stronger associations. The discriminative improvement driven by the CG-BSA formula was superior to that of MDRD4 and CKD-EPI, but remained low overall (increase in C-index ranging from 0.5 to 2%) while not statistically significant in population-based cohorts. The integrated discrimination improvement and net reclassification improvement were higher (*P* < 0.05) for the CG-BSA formula compared to MDRD4 and CKD-EPI in CV risk, HF and post-MI cohorts, but not in population-based cohorts. The CKD-EPI formula was superior overall to MDRD4.

**Conclusions:**

The CG-BSA formula was slightly more accurate in predicting CVM in CV risk, HF, and post-MI cohorts (but not in population-based cohorts). However, the CG-BSA discriminative improvement was globally low compared to MDRD4 and especially CKD-EPI, the latter offering the best compromise between renal function estimation and CVM prediction.

**Electronic supplementary material:**

The online version of this article (doi:10.1186/s12916-016-0731-2) contains supplementary material, which is available to authorized users.

## Background

Renal impairment is a major risk factor for mortality in various populations [1–3], making it an essential risk stratification tool. In certain high-risk populations, renal function is of paramount importance for prognostic purposes in order to establish better and personalized follow-up programs and prognosis-modifying interventions [4]. Hence, risk prediction properties should be considered at least as valuable as the accuracy of renal function quantification per se. Renal function estimation in daily practice is performed by “indirect” parameters (as most accurate gold-standard methods are not practical nor economically suitable for routine use [5]), incorporating variables such as creatinine, age, gender, weight and height through a variety of available formulas. The most commonly used formulas to estimate glomerular filtration rate (eGFR) include the Modification of Diet in Renal Disease (MDRD) formula [6] and the simplified MDRD4 formula [7], both of which have been tested in diverse populations with reproducible results [8], as well as the Chronic Kidney Disease Epidemiology Collaboration (CKD-EPI) equation, which provides the most accurate GFR estimation (compared to a renal-clearance “gold standard”) and is the formula advocated by contemporary consensus [4, 9, 10]. Creatinine clearance (CrCl) estimation can be performed by the Cockcroft–Gault (CG) formula [11]. It should be emphasized that CrCl estimation by the CG formula comprises glomerular CrCl plus tubular CrCl, and therefore the overall CrCl may overestimate GFR by up to 40% in younger individuals without chronic kidney disease [12, 13]. However, in older individuals, the CG formula may underestimate GFR [14, 15]. The CG formula has more recently been modified and validated taking into account body surface area (BSA) [16]. This BSA-adjusted CG formula (CG-BSA) is likely to provide a more accurate estimation of CrCl compared to the original CG formula (hence the use of the CG-BSA formula in the main analysis of this manuscript) [5, 10, 16, 17].

The primary goal of a renal function estimation equation is to estimate GFR (and not tubular clearance). Therefore, for this purpose, the MDRD and CKD-EPI equations are suitable, whereas the CG/CG-BSA equations are not. Nonetheless, in addition to the GFR estimation, prognostic ability is also of utmost importance. Therefore, the aforementioned equations have also been compared regarding their prognostic implications in population-based, cardiovascular (CV) risk, heart failure (HF), and myocardial infarction (MI) populations [3, 18–23]. While extensive information has been provided in these settings using the MDRD4 and CKD-EPI formulas for prognostic and risk estimation purposes with heterogeneous results [19–21], little is known regarding the use of the CG-BSA formula.

The aim of the present study was to conduct a head-to-head comparison of the eGFR and CrCl formulas in terms of prognostic value in four large populations/cohorts, namely population-based, CV risk, HF, and post-MI cohorts.

## Methods

### Study population: the Heart ‘OMics’ in AGEing (HOMAGE) initiative and the high-risk myocardial infarction database initiative

Eligible studies included population studies, patient cohorts, and randomized controlled trials (RCTs). All studies had baseline information on demographic, clinical, and laboratory characteristics and subsequent follow-up reports (cardiovascular mortality was used for the present study). The partners involved in the HOMAGE-consortium contributed data from completed and ongoing studies. These data have been previously published [24] and include patient data derived from (1) population-based cohorts (FLEMENGHO [25] and PREDICTOR [26]); (2) CV risk factor cohorts (ASCOT [27], PROSPER [28], HVC, DYDA [29] and BIOMARCOEURS [30]); and (3) HF cohorts (HULL LIFELAB and TIME-CHF [31]), all of which had baseline information regarding age, sex, weight, height, and plasma creatinine [24].

The High-risk MI Initiative consists of a previously published cohort of pooled patient data derived from four clinical trials [32]. Briefly, the main objectives of the project are to provide a comprehensive and statistically robust description of long-term clinical outcomes in high-risk survivors of MI and to identify the predictors of these different outcomes. In addition, the creation of this large pooled dataset provides statistical power to examine outcomes in important sub-groups of patients defined by co-morbidity and other baseline variables. The datasets included in this pooling initiative were the effect of Carvedilol on Outcome after Myocardial Infarction in Patients with Left Ventricular Dysfunction trial (CAPRICORN) [33, 34], the Eplerenone Post-Acute Myocardial Infarction Heart Failure Efficacy and Survival Study (EPHESUS) [35, 36], the Optimal Trial in Myocardial Infarction with Angiotensin II Antagonist Losartan (OPTIMAAL) [37, 38], and the Valsartan in Acute Myocardial Infarction trial (VALIANT) [39, 40]. Full details of total enrolled patients, the inclusion and exclusion criteria for each trial, the endpoints, and the results have been previously published. Each trial enrolled patients with left ventricular systolic dysfunction, HF, or both between 12 h and 21 days after acute MI.

The respective chairpersons of the Steering Committees of the four trials initiated the pooling project.

The studies were all conducted in accordance with the Declaration of Helsinki and approved by site ethics committees. All participants gave written informed consent to participate in the studies.

### Renal function analyses and estimation of GFR and CrCl

Baseline laboratory measurements were obtained at the time of inclusion and plasma creatinine concentrations were recorded in all studies. The eGFR and CrCl were calculated with the published equations for CKD-EPI [4], the four-variable MDRD4 [6], and CG-BSA [16].

### CKD-EPI

$$ \begin{array}{l}\mathrm{C}\mathrm{K}\mathrm{D}-\mathrm{E}\mathrm{P}\mathrm{I} = 141 \times \min {\left(\mathrm{p}\mathrm{C}\mathrm{r}/\mathrm{k},1\right)}^{\mathrm{a}} \times \max {\left(\mathrm{p}\mathrm{C}\mathrm{r}/\mathrm{k},1\right)}^{-1.209}\kern0.5em  \times 0.{993}^{\mathrm{a}\mathrm{ge}}\kern0.5em  \times \\ {}\left(1.018\ \mathrm{if}\ \mathrm{female}\right) \times \left(1.159\ \mathrm{if}\ \mathrm{black}\right)\end{array} $$

where adjusted serum creatinine was calculated as described below, k is 0.7 for females and 0.9 for males, a is −0.329 for females and −0.411 for males, min indicates the minimum of serum creatinine adjusted/k or 1, and max indicates the maximum of adjusted serum creatinine/k or l.

### MDRD4

$$ \mathrm{MDRD}4 = 186 \times {\mathrm{pCr}}^{-1.154}\kern0.5em  \times {\mathrm{age}}^{-0.203}\kern0.5em  \times \left(0.742\ \mathrm{if}\ \mathrm{female}\right) \times \left(1.210\ \mathrm{if}\ \mathrm{black}\right) $$

### CG-BSA

$$ \begin{array}{l}\mathrm{C}\mathrm{G}-\mathrm{B}\mathrm{S}\mathrm{A} = \left[\left(140\ \hbox{--}\ \mathrm{age}\right) \times \mathrm{weight}\ \mathrm{in}\ \mathrm{kg} \times \left(0.85\ \mathrm{if}\ \mathrm{female}\right)\right]\ /\ \left(72 \times \mathrm{p}\mathrm{C}\mathrm{r}\ \mathrm{in}\ \mathrm{mg}/\mathrm{dL}\right) = \\ {}\mathrm{C}\mathrm{ockcroft}-\mathrm{Gault}\end{array} $$

followed by (1.73 m^2^ × GFR-CG)/BSA of the patient; where the GFR-CG is in milliliters per minute and the BSA is calculated in meters squared using the Mosteller equation [41].

Patients were also classified into eGFR categories 1–5: Stage 1, eGFR ≥ 90; Stage 2, eGFR ≥ 60 and < 90; Stage 3a, eGFR ≥ 45 and < 60; Stage 3b, eGFR ≥ 30 and < 45; Stage 4, eGFR ≥ 15 and < 30; Stage 5, eGFR < 15.

### Outcomes

The present study analyzed cardiovascular mortality (CVM) as the primary outcome. Endpoints were independently adjudicated in the respective trials and cohorts.

### Statistical methods

In descriptive analyses, continuous variables are expressed as mean ± standard deviation (SD) if normally distributed or as median (percentile_25–75_) if skewed. Categorical variables are expressed as frequencies and proportions (%).

The one-way analysis of variance “ANOVA test” was used to compare the renal function estimates obtained with the MDRD4, CKD-EPI, and CG-BSA formulas.

Univariable time-to-event comparisons were performed using the log-rank test and survival was estimated with the Kaplan–Meier method. Cox proportional hazard regression models were used to model long-term survival as a function of the formulas both in univariable and multivariable analysis. Cox models assumptions were verified and GFR/CrCl formulas were converted to restricted cubic splines to overcome linearity issues. An interaction term between the variable of interest and time was tested within the Cox model. In the multivariable models, the covariates were chosen from demographic (age and sex) and clinical (smoking, hypertension, diabetes, heart rate, and systolic blood pressure) parameters that were previously found to be clinically relevant and previously reported [42]. A significant “interaction” between age and “renal function” formulas was present in various populations; thus, these results are presented for age subgroups for which the “age” variable was not included in the adjustment models in order to decrease model instability. Left ventricular ejection fraction and hemoglobin were also not included in the “adjusted” models due to a high (>7 5%) percentage of missing values.

Correlation estimates were verified prior to modeling and correlation coefficients < 0.6 were considered to rule out noteworthy multicollinearity within the survival models [43].

To assess the relative importance and discriminative value of renal function estimators in terms of outcome, Harrell’s c-index [44] was evaluated and compared with the correlated c-indices using the approach proposed by Kang et al. [45].

Calibration was assessed visually by plotting the mean of model-predicted survival at 2 years in each decile of predicted survival against the observed survival estimated by the Kaplan–Meier method. The higher discriminative value associated with the “net reclassification improvement” (NRI) was assessed at 2 years [46, 47]. This method assesses the ability of a new model to reclassify subjects with and without a clinical event during follow-up. The ability of the new model to reclassify is summarized by the NRI statistic. As previously used by our team [48], the continuous NRI method developed by Uno [47] and implemented in the survIDINRI package of the R software (The R Foundation for Statistical Computing) was used. The continuous NRI method does not require a prior definition of strata risk, thus considering the change in the estimation prediction as a continuous variable. The integrated discrimination improvement (IDI) for each eGFR and CrCl estimator was also calculated. The IDI evaluates the difference between the integrated sensitivity gain and the integrated specificity loss due to the addition of the eGFR and CrCl estimator to the prognostic model.

Statistical analyses were performed using SPSS 23 software (IBM Corp. Released 2013. IBM SPSS Statistics for Windows, Version 23.0. Armonk, NY: IBM Corp.) and the R software (The R Foundation for Statistical Computing).

A *P* value of less than 0.05 was considered statistically significant.

## Results

### Population characteristics and renal function stage classification

A total of 2644 patients were analyzed in population-based cohorts, 20,895 in CV risk cohorts, 1801 in HF populations, and 28,771 in post-MI cohorts.

The mean ± SD age was 66.4 ± 11.5, 66.7 ± 9.4, 73.7 ± 10.4, and 65 ± 11.5 years in the population-based, CV risk, HF, and post-MI populations, respectively. As expected, population-based cohorts had higher eGFR/CrCl while HF populations had lower eGFR/CrCl (as calculated by all formulas). Concordantly, the proportion of CVM was also lower in population-based cohorts while increasing progressively in CV risk, post-MI populations, and HF cohorts (2.0% vs. 3.8% vs. 15.3% vs. 15.7%, respectively; *P* < 0.001). The baseline characteristics of all populations are summarized in Table 1.

**Table 1:** Baseline characteristics of the different cohorts

Variables	Population-based	Cardiovascular risk	Heart failure	Post-myocardial infarction	*P* value
Number of patients	2644	20,895	1801	28,771	<0.001
Demographic					
Age, years	66.4 ± 14.1	66.7 ± 9.4	73.7 ± 10.4	65 ± 11.5	<0.001
Male sex, n (%)	1359 (51.4)	14,083 (67.4)	1093 (60.7)	20,189 (70.2)	<0.001
Smokers, n (%)	400 (15.1)	5994 (28.8)	185 (10.7)	18,235 (63.4)	<0.001
Clinical and echocardiographic					
Hypertension, n (%)	1397 (53.1)	17,892 (85.6)	861 (47.8)	15,570 (54.1)	<0.001
Diabetes, n (%)	334 (12.7)	5164 (24.7)	541 (30.1)	7386 (25.7)	<0.001
SBP, mmHg	136 ± 18	159 ± 21	133 ± 24	122 ± 17	<0.001
Heart rate, bpm	67 ± 12	71 ± 13	75 ± 15	76 ± 13	<0.001
LVEF, %^a^	67 ± 8	56 ± 13	41 ± 15	34 ± 9	<0.001
Height, m	1.67 ± 8.9	1.69 ± 9.5	1.66 ± 9.7	1.69 ± 9.4	<0.001
Weight, kg	73.8 ± 13.9	80.7 ± 15.8	76.4 ± 18.2	79.1 ± 15.5	<0.001
Laboratory					
Hemoglobin, g/dL^a^	13.9 ± 1.3	13.4 ± 1.9	13.2 ± 1.8	13.3 ± 1.7	<0.001
Creatinine, mg/dL	0.96 ± 0.24	1.12 ± 0.25	1.24 ± 0.53	1.12 ± 0.32	<0.001
MDRD4, mL/min/1.73 m^2^	77.1 ± 18.2	66.8 ± 15.3	63.3 ± 24.4	71.05 ± 39.61	<0.001
CKD-EPI, mL/min/1.73 m^2^	74.6 ± 17.0	65.2 ± 15.4	59.3 ± 21.9	67.93 ± 20.75	<0.001
CG-BSA, mL/min/1.73 m^2^	71.1 ± 20.6	64.6 ± 17.8	57.2 ± 25.5	68.32 ± 32.89	<0.001
Follow-up and events					
Follow-up, years	4.2 (3.6–4.9)	5.0 (3.3–5.9)	2.6 (1.2–3.5)	1.0 (0.2–1.9)	<0.001
CVM, n (%)	52 (2.0)	785 (3.8)	283 (15.7)	4400 (15.3)	<0.001

*N* number, *MI* myocardial infarction, *MV* missing values, *SBP* systolic blood pressure, *LVEF* left ventricular ejection fraction, *CVM* cardiovascular mortality, *CG-BSA* Cockcroft–Gault formula adjusted for body surface area, *MDRD4* modification of diet in renal disease-4 formula, *CKDEPI* Chronic Kidney Disease Epidemiology Collaboration equation

Normally distributed variables are presented as mean ± standard deviation; skewed variables are presented as median (percentile 25–75); proportions are presented as absolute numbers (n) and proportions (%). *P* values for “between group differences” were determined by one-way analysis of variance (ANOVA) tests

^a^These variables present > 75% missing values in all populations, therefore they are not included in the adjusted models

The CG-BSA formula reclassified a higher proportion of patients as having worse renal function (“stages ≥ 3”) in all populations. On the other hand, the proportion of CVM events (relative to the number of patients) was higher overall using the MDRD4 formula. For example, in population-based cohorts, patients reclassified into worse renal function stages experienced 6.9% of events according to the CG-BSA formula, 6.5% of events with CKD-EPI, and 7.2% of events with MDRD4 (similar observations were found in the other cohorts) (Table 2).

**Table 2:** Proportion of patients within “renal function” categories according to the different formulas

Formula	MDRD4		CKD-EPI		CG-BSA		*P* value
RF stage	Patients, n (%)	Events, n (%)	Patients, n (%)	Events, n (%)	Patients, n (%)	Events, n (%)	
Population-based							
Stage 1: ≥ 90	521 (19.7)	9 (1.7)	425 (16.1)	3 (0.7)	429 (16.2)	4 (0.9)	<0.001
Stage 2: 60–89	1688 (63.8)	26 (1.5)	1701 (64.3)	29 (1.7)	1410 (53.3)	17 (1.2)	<0.001
Stage 3a: 45–59	353 (13.3)	11 (3.1)	395 (14.9)	12 (3.0)	602 (22.8)	17 (2.8)	<0.001
Stage 3b/4/5: < 45	83 (3.1)	6 (7.2)	123 (4.7)	8 (6.5)	203 (7.7)	14 (6.9)	<0.001
Cardiovascular risk							
Stage 1: ≥ 90	1223 (5.9)	27 (2.2)	1165 (5.6)	20 (1.7)	1727 (8.3)	28 (1.6)	<0.001
Stage 2: 60–89	12,992 (62.2)	388 (3.0)	11,956 (57.2)	335 (2.8)	10,284 (49.2)	253 (2.4)	<0.001
Stage 3a: 45–59	5325 (25.5)	242 (4.5)	5845 (28.0)	261 (4.5)	6277 (30.0)	293 (4.7)	<0.001
Stage 3b/4/5: < 45	1355 (6.5)	128 (9.4)	1929 (9.2)	169 (8.8)	2607 (12.5)	211 (8.1)	<0.001
Heart failure							
Stage 1: ≥ 90	229 (12.7)	18 (7.8)	165 (9.2)	7 (4.2)	179 (9.9)	4 (2.2)	<0.001
Stage 2: 60–89	738 (41.0)	77 (10.4)	685 (38.0)	70 (10.2)	533 (29.6)	45 (8.4)	<0.001
Stage 3a: 45–59	405 (22.5)	71 (17.5)	428 (23.8)	63 (14.7)	443 (24.6)	62 (14.0)	<0.001
Stage 3b/4/5: < 45	429 (23.8)	117 (27.3)	523 (29.0)	143 (27.3)	646 (35.9)	172 (26.6)	<0.001
Post-myocardial infarction							
Stage 1: ≥ 90	4277 (15.3)	405 (9.5)	4210 (15.1)	351 (8.3)	4315 (15.8)	337 (7.8)	<0.001
Stage 2: 60–89	14,328 (51.4)	1670 (11.7)	13,400 (48.1)	1515 (11.3)	11,746 (43.1)	1197 (10.2)	<0.001
Stage 3a: 45–59	6261 (22.5)	1247 (19.9)	6453 (23.1)	1228 (19.0)	6746 (24.8)	1219 (18.1)	<0.001
Stage 3b: 30–45	2622 (9.4)	793 (30.2)	3174 (11.4)	924 (29.1)	3744 (13.7)	1090 (29.1)	<0.001
Stage 4/5: < 30	388 (1.5)	162 (41.8)	639 (2.4)	259 (40.5)	682 (2.6)	280 (41.1)	<0.001

*RF* renal function, *CG-BSA* Cockcroft-Gault formula adjusted for body surface area, *MDRD4* modification of diet in renal disease-4 formula, *CKD-EPI* Chronic Kidney Disease Epidemiology Collaboration equation

Events refer to cardiovascular mortality

### Mortality prediction and accuracy

The three formulas were effective with regard to CVM prediction. However, important differences were notably observed between populations and formulas.

**Fig. 1:**
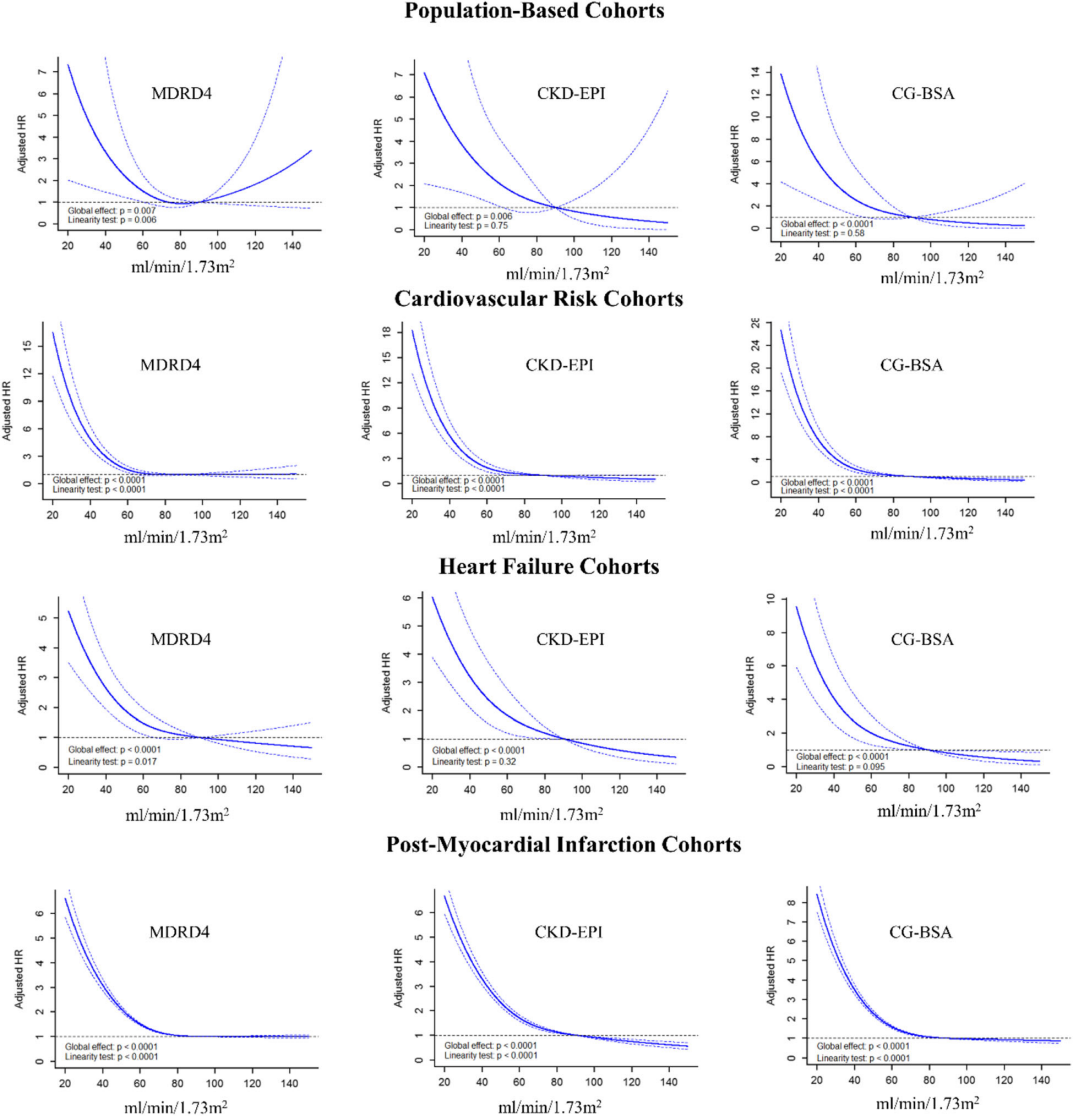
Association between “renal function” formulas and cardiovascular mortality in each population setting using restricted cubic splines. CG-BSA, Cockcroft–Gault formula adjusted for body surface area; MDRD4_,_ Modification of Diet in Renal Disease-4 Formula; CKD-EPI_,_ Chronic Kidney Disease Epidemiology Collaboration equation. Models adjusted for sex, smoking status, hypertension history, diagnosis of diabetes, heart rate, and systolic blood pressure

Associations between the various “renal function” formulas and CVM within the studied populations are demonstrated in Table 3 for categorical variables and in Fig. 1 for “cubic spline transformed” continuous variables (linearity tests are shown in Additional file 1: Table S1). As continuous variables, all formulas showed to be independently associated with CVM in the various cohorts. An eGFR/CrCl lower than 60 mL/min/1.73 m^2^ was associated with an increase in event rate in all cohorts; however, the less accurate (wide confidence intervals) associations in population-based cohorts reflect the low CVM event rate in this setting (Fig. 1 and Additional file 1: Figure S1 for “log-transformed” hazard ratios). In population-based cohorts, the “categorical” MDRD4 formula lost its predictive value after adjustment for clinically relevant confounders (sex, smoking, hypertension, diabetes, systolic blood pressure, and heart rate), whereas the “categorical” CKD-EPI and CG-BSA formulas remained significant for the lower renal function stages as compared to stage 1 (reference category) [HR for CKD-EPI stages 3b/4/5 = 6.43 (95% CI, 1.61–25.65), *P* = 0.008 and HR for CG-BSA stages 3b/4/5 = 5.35 (95% CI, 1.66–17.17), *P* = 0.005]. In CV risk, HF, and post-MI populations, all “categorical” formulas were found to be independently associated with CVM for the lower “renal function” stages with intersecting confidence intervals (Table 3).

**Table 3:** Cox-regression models for cardiovascular mortality according to the different formulas and cohorts

Variables	Univariable: HR (95% CI)	*P* value	Adjusted: HR (95% CI)	*P* value
Population-based				
MDRD4				
Categorical		0.001		0.046
Stage 1: ≥ 90	Reference		Reference	
Stage 2: 60–89	0.818 (0.382–1.751)	0.605	0.799 (0.370–1.726)	0.568
Stage 3a: 45–59	1.908 (0.790–4.609)	0.151	1.423 (0.563–3.597)	0.456
Stage 3b/4/5: < 45	4.624 (1.644–13.008)	0.004	3.004 (0.976–9.247)	0.055
CKD-EPI				
Categorical		<0.001		0.019
Stage 1: ≥ 90	Reference		Reference	
Stage 2: 60–89	2.471 (0.752–8.117)	0.136	2.007 (0.607–6.640)	0.254
Stage 3a: 45–59	4.968 (1.400–17.635)	0.013	3.122 (0.851–11.456)	0.086
Stage 3b/4/5: < 45	11.207 (2.967–42.335)	<0.001	6.426 (1.610–25.652)	0.008
CG-BSA				
Categorical		<0.001		<0.001
Stage 1: ≥ 90	Reference		Reference	
Stage 2: 60–89	1.367 (0.459–4.070)	0.574	1.078 (0.360–3.230)	0.894
Stage 3a: 45–59	3.658 (1.226–10.915)	0.020	2.393 (0.787–7.275)	0.124
Stage 3b/4/5: < 45	9.562 (3.131–29.199)	<0.001	5.347 (1.665–17.170)	0.005
Cardiovascular risk				
MDRD4				
Categorical		<0.001		<0.001
Stage 1: ≥ 90	Reference		Reference	
Stage 2: 60–89	1.008 (0.682–1.489)	0.969	1.096 (0.740–1.624)	0.647
Stage 3a: 45–59	1.682 (1.130–2.504)	0.010	1.924 (1.285–2.881)	0.001
Stage 3b/4/5: < 45	4.308 (2.844–6.526)	<0.001	5.138 (3.361–7.857)	<0.001
CKD-EPI				
Categorical		<0.001		<0.001
Stage 1: ≥ 90	Reference		Reference	
Stage 2: 60–89	1.292 (0.823–2.029)	0.266	1.391 (0.884–2.190)	0.154
Stage 3a: 45–59	2.263 (1.436–3.566)	<0.001	2.543 (1.605–4.029)	<0.001
Stage 3b/4/5: < 45	5.397 (3.393–8.583)	<0.001	6.292 (3.923–10.091)	<0.001
CG-BSA				
Categorical		<0.001		<0.001
Stage 1: ≥ 90	Reference		Reference	
Stage 2: 60–89	1.347 (0.911–1.990)	0.135	1.504 (1.009–2.241)	0.045
Stage 3a: 45–59	2.927 (1.986–4.315)	<0.001	3.416 (2.290–5.094)	<0.001
Stage 3b/4/5: < 45	6.277 (4.227–9.322)	<0.001	7.520 (4.981–11.353)	<0.001
Heart failure				
MDRD4				
Categorical		<0.001		<0.001
Stage 1: ≥ 90	Reference		Reference	
Stage 2: 60–89	1.076 (0.643–1.800)	0.781	1.016 (0.605–1.706)	0.952
Stage 3a: 45–59	1.791 (1.066–3.007)	0.028	1.517 (0.896–2.569)	0.121
Stage 3b/4/5: < 45	3.298 (2.007–5.420)	<0.001	2.824 (1.704–4.680)	<0.001
CKD-EPI				
Categorical		<0.001		<0.001
Stage 1: ≥ 90	Reference		Reference	
Stage 2: 60–89	2.194 (1.008–4.774)	0.048	2.248 (1.030–4.906)	0.042
Stage 3a: 45–59	2.997 (1.372–6.549)	0.006	2.871 (1.306–6.310)	0.009
Stage 3b/4/5: < 45	6.557 (3.070–14.006)	<0.001	5.784 (2.692–12.430)	<0.001
CG-BSA				
Categorical		<0.001		<0.001
Stage 1: ≥ 90	Reference		Reference	
Stage 2: 60–89	3.243 (1.164–9.031)	0.024	3.457 (1.240–9.642)	0.018
Stage 3a: 45–59	5.266 (1.915–14.485)	0.001	5.078 (1.838–14.024)	0.002
Stage 3b/4/5: < 45	12.388 (4.595–33.398)	<0.001	11.006 (4.066–29.793)	<0.001
Post-myocardial infarction				
MDRD4				
Categorical		<0.001		<0.001
Stage 1: ≥ 90	Reference		Reference	
Stage 2: 60–89	1.243 (1.115–1.385)	<0.001	1.296 (1.162–1.446)	<0.001
Stage 3a: 45–59	2.253 (2.014–2.521)	<0.001	2.251 (2.009–2.523)	<0.001
Stage 3b: 30–45	3.692 (3.276–4.162)	<0.001	3.509 (3.104–3.967)	<0.001
Stage 4/5: < 30	5.509 (4.591–6.610)	<0.001	5.076 (4.208–6.123)	<0.001
CKD-EPI				
Categorical		<0.001		<0.001
Stage 1: ≥ 90	Reference		Reference	
Stage 2: 60–89	1.363 (1.214–1.531)	<0.001	1.427 (1.269–1.603)	<0.001
Stage 3a: 45–59	2.430 (2.158–2.736)	<0.001	2.442 (2.165–2.754)	<0.001
Stage 3b: 30–45	3.976 (3.516–4.497)	<0.001	3.852 (3.396–4.368)	<0.001
Stage 4/5: < 30	6.098 (5.193–7.160)	<0.001	5.765 (4.887–6.800)	<0.001
CG-BSA				
Categorical		<0.001		<0.001
Stage 1: ≥ 90	Reference		Reference	
Stage 2: 60–89	1.302 (1.154–1.470)	<0.001	1.369 (1.213–1.546)	<0.001
Stage 3a: 45–59	2.447 (2.169–2.761)	<0.001	2.523 (2,234–2.849)	<0.001
Stage 3b: 30–45	4.230 (3.744–4.780)	<0.001	4.239 (3.745–4.799)	<0.001
Stage 4/5: < 30	6.536 (5.578–7.659)	<0.001	6.639 (5.649–7.802)	<0.001

Adjusted model for sex, smoking status, history of hypertension, diagnosis of diabetes, heart rate, and systolic blood pressure (not adjusted for hemoglobin or left ventricular ejection fraction due to > 75% of missing values in all datasets)

*CG-BSA* Cockcroft–Gault formula adjusted for body surface area, *MDRD4* Modification of Diet in Renal Disease-4 formula, *CKD-EPI* Chronic Kidney Disease Epidemiology Collaboration equation

The associations of GFR/CrCl formulas with cardiovascular mortality are mostly non-linear. In concordance, these results are presented as restricted cubic splines in Fig. 1. Interactions, linearity and colinearity were verified and excluded at each model step.

**Fig. 2:**
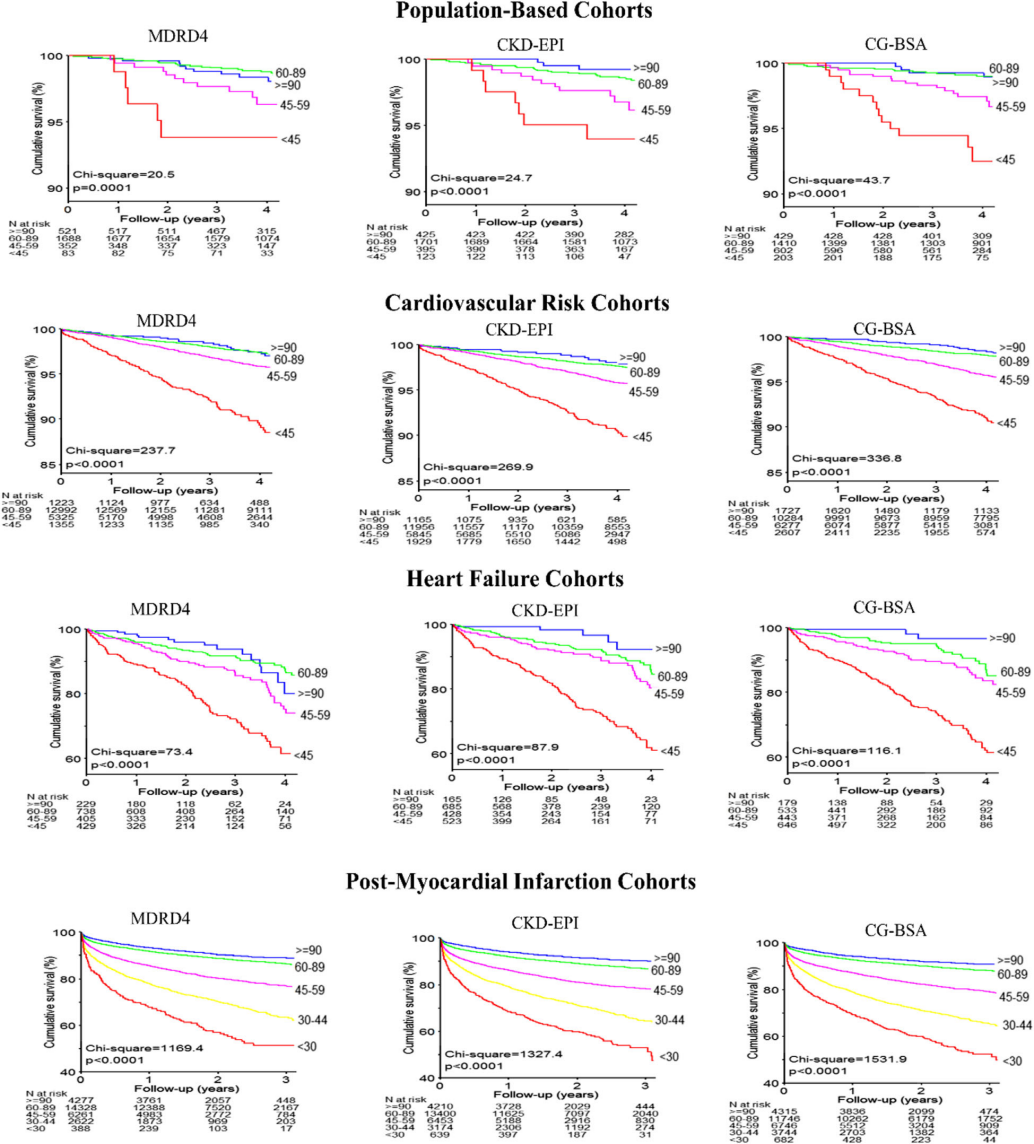
Kaplan–Meier curves for cardiovascular mortality according to the different formulas and populations. CG-BSA, Cockcroft–Gault formula adjusted for body surface area; MDRD4_,_ Modification of Diet in Renal Disease-4 formula; CKD-EPI_,_ Chronic Kidney Disease Epidemiology Collaboration equation

Long-term survival Kaplan–Meier curves are shown in Fig. 2. All formulas showed highly significant predictive prognostic values (log-rank test, *P* < 0.001). However, eGFR/CrCl stages diverged in a more pronounced manner with the CG-BSA formula in all populations (higher χ^2^ values) (Fig. 2).

Significant “interactions” were frequently observed between age and the eGFR/CrCl formulas, suggesting that renal function is a more relevant prognosticator in younger populations (Additional file 1: Table S2).

### Discrimination, calibration, and reclassification improvement analysis

In the CV risk, HF, and post-MI cohorts, the CG-BSA formula demonstrated statistically superior discriminative capacity (c-statistics) when added on top of a prognostic model (including gender, smoking, hypertension, diabetes status, heart rate and systolic blood pressure) as well as when compared to the GFR formulas (on top of the same model). Despite being statistical significant, the discriminative improvement driven by the CG-BSA formula was globally low (vs. eGFR formulas) ranging from 0.5 to 2% (Table 4). In population-based cohorts, the discriminative capacity improvement was not statistically significant, nor superior to the eGFR formulas (*P* ≥ 0.05 for all comparisons). The discriminative capacity of the CKD-EPI formula was superior to that of the MDRD4 formula in CV risk, HF, and post-MI cohorts, but not in population-based cohorts (Table 4).

**Table 4:** C-statistics, integrated discrimination improvement, and net reclassification improvement of restricted cubic spline “renal function” formulas for cardiovascular mortality discrimination and reclassification in the studied cohorts

Cohorts	Model comparison (A vs. B)	C-index A%	C-index B%	ΔC-index%	*P* value	IDI% (95% CI)	*P* value	NRI% (95% CI)	*P* value
Population	Model vs. MDRD4	72.8	75.7	2.9 (−0.3 to 6.1)	0.072	0.5 (−0.1 to 4.3)	0.16	16.7 (−2.0 to 44.1)	0.072
	Model vs. CKD-EPI	72.8	75.2	2.4 (−1.6 to 6.4)	0.23	0.6 (0.1 to 4.8)	0.016	23.4 (−0.9 to 44.4)	0.054
	Model vs. CG-BSA	72.8	77.3	4.5 (−0.7 to 9.7)	0.087	1.0 (0.2 to 5.7)	0.020	39.1 (4.6 to 51.2)	0.020
	MDRD4 vs. CKD-EPI	75.7	75.2	0.5 (−2.4 to 3.4)	0.74	0.2 (0.0 to 0.8)	0.026	23.2 (−10.7 to 36.8)	0.20
	MDRD4 vs. CG-BSA	75.7	77.3	1.6 (−2.8 to 6.1)	0.48	0.5 (0.0 to 2.1)	0.045	27.4 (−6.8 to 47.9)	0.13
	CKD-EPI vs. CG-BSA	75.2	77.3	2.1 (−0.1 to 4.4)	0.067	0.4 (−0.2 to 1.6)	0.14	31.3 (−13.9 to 48.1)	0.17
CV risk	Model vs. MDRD4	58.9	66.2	7.3 (5.2 to 9.5)	<0.001	0.9 (0.6 to 1.6)	<0.001	18.5 (13.0 to 24.3)	<0.001
	Model vs. CKD-EPI	58.9	67.5	8.6 (6.4 to 10.9)	<0.001	1.1 (0.7 to 1.7)	<0.001	20.3 (15.3 to 25.8)	<0.001
	Model vs. CG-BSA	58.9	70.2	11.3 (8.9 to 13.7)	<0.001	1.5 (1.0 to 2.4)	<0.001	27.3 (21.4 to 32.6)	<0.001
	MDRD4 vs. CKD-EPI	66.2	67.5	1.3 (0.8 to 1.8)	<0.001	0.1 (0.1 to 0.2)	0.006	23.0 (16.7 to 29.5)	<0.001
	MDRD4 vs. CG-BSA	66.2	70.2	4.0 (2.7 to 5.3)	<0.001	0.6 (0.3 to 0.9)	<0.001	22.8 (16.8 to 29.1)	<0.001
	CKD-EPI vs. CG-BSA	67.5	70.2	2.7 (1.7 to 3.7)	<0.001	0.4 (0.2 to 0.7)	<0.001	21.1 (14.6 to 27.6)	<0.001
Heart failure	Model vs. MDRD4	70.7	75.0	4.2 (1.9 to 6.5)	<0.001	2.7 (1.2 to 4.8)	<0.001	24.6 (15.9 to 34.1)	<0.001
	Model vs. CKD-EPI	70.7	75.5	4.7 (2.3 to 7.1)	<0.001	3.0 (1.5 to 5.2)	<0.001	25.5 (15.5 to 34.1)	<0.001
	Model vs. CG-BSA	70.7	77.0	6.3 (3.6 to 8.9)	<0.001	4.4 (2.6 to 7.2)	<0.001	29.6 (20.1 to 39.9)	<0.001
	MDRD4 vs. CKD-EPI	75.0	75.5	0.5 (0.2 to 0.8)	0.003	0.3 (0.1 to 0.5)	0.006	12.7 (1.6 to 23.8)	0.024
	MDRD4 vs. CG-BSA	75.0	77.0	2.1 (1.1 to 3.0)	<0.001	1.7 (0.8 to 2.7)	<0.001	24.9 (14.7 to 34.7)	<0.001
	CKD-EPI vs. CG-BSA	75.5	77.0	1.6 (0.8 to 2.4)	<0.001	1.4 (0.6 to 2.3)	<0.001	26.5 (14.4 to 34.5)	<0.001
Post-MI	Model vs. MDRD4	61.7	66.5	4.9 (4.2 to 5.6)	<0.001	3.4 (2.9 to 3.9)	<0.001	21.1 (19.4 to 22.8)	<0.001
	Model vs. CKD-EPI	61.7	67.3	5.6 (4.9 to 6.4)	<0.001	3.8 (3.4 to 4.4)	<0.001	22.4 (20.6 to 24.0)	<0.001
	Model vs. CG-BSA	61.7	68.8	7.1 (6.3 to 7.9)	<0.001	4.9 (4.3 to 5.6)	<0.001	24.9 (23.0 to 26.5)	<0.001
	MDRD4 vs. CKD-EPI	66.5	67.3	0.8 (0.6 to 0.9)	<0.001	0.5 (0.4 to 0.6)	<0.001	16.4 (13.6 to 20.0)	<0.001
	MDRD4 vs. CG-BSA	66.6	68.8	2.3 (1.9 to 2.6)	<0.001	1.6 (1.3 to 1.9)	<0.001	20.9 (18.9 to 23.1)	<0.001
	CKD-EPI vs. CG-BSA	67.3	68.8	1.5 (1.3 to 1.8)	<0.001	1.1 (0.9 to 1.4)	<0.001	16.6 (13.8 to 20.4)	<0.001

*CG-BSA* Cockcroft-Gault formula adjusted for body surface area, *MDRD4* modification of diet in renal disease-4 formula, *CKD-EPI* Chronic Kidney Disease Epidemiology Collaboration equation, *CV* cardiovascular, *MI* myocardial infarction, *IDI* integrated discrimination improvement, *NRI* net reclassification improvement, *Δ* delta (change)

All models adjusted for gender, smoking status, history of hypertension, diagnosis of diabetes, heart rate and systolic blood pressure (not adjusted for hemoglobin or left ventricular ejection fraction due to > 75% of missing values in all datasets)

All “renal function” formulas are analyzed using restricted cubic splines

The prognostic models were well calibrated, with intersecting predicted risks and confidence intervals of observed risks in all populations and formulas (Additional file 1: Figure S2). The IDI and NRI were higher (*P* < 0.05) for the CG-BSA formula as compared to MDRD4 and CKD-EPI in CV risk, HF, and post-MI cohorts. In the population-based cohorts, CG-BSA was not superior to the other formulas. The CKD-EPI formula was globally superior to MDRD4 (Table 4).

## Discussion

### General interpretation

The present study showed that CG-BSA was slightly more precise and accurate in terms of CVM prediction in CV risk, HF, and post-MI cohorts, but not in population-based cohorts, followed by the CKD-EPI formula. The CG-BSA formula estimates CrCl (and not the GFR contrary to the MDRD4 and CKD-EPI formulas) and requires individual height and weight for its computation. Therefore, CKD-EPI offers the best compromise between renal function estimation and CVM prediction.

### Development and validation of GFR and CrCl formulas

Estimation of GFR (and CrCl in the case of CG/CG-BSA formulas) is an inexpensive, practical, and fairly reliable means to assess renal function in clinical practice [5]. However, certain drawbacks need to be considered since creatinine-based eGFR/CrCl is dependent on multiple factors including age, sex, race, diet, muscle mass, tubular secretion, unstable renal function, and BSA [16]. Hence, the varying performance of the equations is likely to rely on their “core formula” development and validation.

The equation proposed by Cockcroft and Gault in 1976 was developed in a Caucasian male population of 236 patients aged 18–92 years in order to predict CrCl (and not GFR) in situations in which renal function was only slightly impaired [11]. This original CG formula was found to be inaccurate for GFR prediction. The CG formula incorporates glomerular CrCl plus tubular CrCl, resulting in an overestimation of GFR in younger healthy populations [12, 13], whereas in older populations, it may underestimate GFR due to its formula computation, i.e., the numerator includes “140-age”; thus, for the same weight and creatinine, a very old patient will have a disproportionate underestimation of their GFR [14, 15]. Despite the fact that CG adjusted for BSA may improve its accuracy [16, 49, 50], it should be emphasized that neither the original CG nor the BSA-adjusted formula should be used for GFR estimation and that these formulas are not recommended by updated guidelines. Still, many clinicians and laboratories continue to use the latter on a daily basis [10]. In the late nineties, the MDRD group developed models that improved the prediction of eGFR from plasma creatinine concentration [6], providing more reliable estimations of kidney function than the CG-BSA formula [51]. Finally, the CKD-EPI equation was developed from a population of more than 16,000 participants. This formula was validated and found to be more accurate than the MDRD4 formula (compared to a renal-clearance “gold standard”), and thus proposed as a first choice to estimate GFR in routine clinical practice [4, 9].

It should also be noted that the MDRD and CKD-EPI equations allow GFR estimation without the need of available “individualized” weight or height (as most laboratories do not assess these data), this information being standardized in the formulas’ “intrinsic design” [52].

Comparing eGFR/CrCl formulas to a renal-clearance “gold-standard” in large populations clearly underscores the lack of precision of all these formulas [51, 52]. Additionally, the eGFR/CrCl formulas are prone to high misclassification rates (≥30%) of patients according to the Kidney Disease Outcomes Quality Initiative Chronic Kidney Disease (K/DOQI-CKD) classification stages [51–53].

Still, the CKD-EPI formula outperforms the MDRD4 and CG/CG-BSA in terms of eGFR precision and classification in several populations (compared to a “gold standard”) [52], although this is not necessarily the case for outcomes prediction as discussed below.

### Estimation of GFR and CrCl versus mortality prediction

GFR estimation serves not only to estimate renal function (for which purpose the CKD-EPI formula is the most accurate to date), but also to estimate the risk of major outcomes, such as cardiovascular mortality, since renal function is the strongest mortality predictor in many populations [2, 3, 5]. In this regard, the CKD-EPI formula was also found to be superior to MDRD4 for mortality prediction in large general population cohorts, high vascular risk cohorts, and chronic kidney disease cohorts [3]. Specifically, a meta-analysis by Matsushita et al. [3] evaluated the risk implications of the CKD-EPI formula as compared to the MDRD equation in populations comprising 1,130,472 adults from 25 general populations, 7 high-risk (of vascular disease), and 13 CKD cohorts. In this latter study, the CKD-EPI equation reclassified fewer individuals as CKD and more accurately categorized the risk for mortality and ESRD than did the MDRD equation. Nonetheless, the CG-BSA formula has not been consistently used for risk prediction purposes in these population settings, since it does not estimate GFR [3, 22, 23, 54], with the exception of a recent study comparing CG-BSA, CKD-EPI, and MDRD4 in 925 ambulatory HF patients where the CG-BSA formula also showed superiority for mortality risk prediction [19]. Our study distinguishes from previous reports by using the CG formula adjusted for BSA, and reveals a slight superiority of CG-BSA over CKD-EPI and MDRD4 formulas in predicting cardiovascular mortality in the studied CV risk, HF, and post-MI cohorts, but not in population-based cohorts. The CKD-EPI formula also showed good accuracy for CVM prediction and was globally superior to the MDRD4 formula. A possible explanation for the mild improvement in predictive value of the CG-BSA formula, as compared to the MDRD4 and CKD-EPI formulas, is the use of “individually observed” BSA in the CG-BSA formula computation versus “intrinsic design” BSA in the MDRD4 and CKD-EPI formulas, as “individual” BSA carries important prognostic information per se [55]. While the addition of BSA on top of CG-BSA did not improve reclassification indices in an exploratory analysis, these indices were nonetheless improved when BSA was added to MDRD4 and CKD-EPI, supporting that the “individual” BSA can provide prognostic information on top of the “intrinsic design” BSA of MDRD and CKD-EPI (Additional file 1: Table S3) [52, 55]. Another potential explanation is that all of the studied cohorts herein consisted of elderly populations, in which both the CG and CG-BSA are likely to underestimate renal function, as previously highlighted, thereby “reclassifying” more patients into worse renal function categories (as demonstrated in Table 2). However, the CKD-EPI formula provides more accurate renal function estimations (as also discussed above) as well as good CVM prediction. Moreover, it does not require individual height or weight, making this formula easy to implement and favoring its widespread use. Despite the statistically superior discriminative capacity of CG-BSA (as compared to the other formulas) in CV risk, HF, and post-MI cohorts, it is unlikely to be clinically relevant, with CKD-EPI offering the best compromise between renal function estimation and CVM prediction/risk assessment.

### Innovation and clinical and research implications

Globally, the greater accuracy of the CG-BSA equation for CVM prediction has the potential to slightly improve prognostic information in CV risk, HF, and post-MI populations [4]. However, the CG-BSA does not accurately estimate renal function and is only slightly superior statistically (and clinically irrelevant) comparatively to CKD-EPI, which remains the best formula overall.

### Limitations

Several limitations of this study should be acknowledged. First, eGFR/CrCl was tested for cardiovascular mortality prediction and not for GFR accuracy, and therefore the best formula to estimate “actual” GFR cannot be derived from this study. Second, data regarding cystatin-C or microalbuminuria levels were unavailable; these data would have likely enhanced risk prediction models since non-GFR mechanisms (such as microalbuminuria) are also associated with prognosis and models incorporating both GFR and microalbuminuria perform more accurately in prognosis prediction [56]. Third, in a small number of patients, the fact that the reference weight at admission used for the CG formula may not have been the true dry weight and thus potentially overestimated cannot be disregarded. Fourth, creatinine was measured at baseline, whereas the study did not account for “time-dependent” variations that could also have major prognostic implications. Fifth, creatinine measurements were not “standardized” between different populations and cohorts; however, this potential heterogeneity in creatinine values was not systematic, reinforcing the strength of our results. Sixth, in the computation of CG-BSA, height and weight were added in an individual basis, it is thus necessary for these data to be available (limiting a wider applicability of this formula). Finally, the populations included in the studied cohorts all had a mean age above 65 years such that the CG formula (independently of BSA adjustment) is likely to underestimate renal function (as described in the discussion section) and thus reclassify a higher proportion of patients into lower renal function stages; consequently, these data may not be replicable in younger populations.

## Conclusion

The CG-BSA formula was the most accurate in predicting CVM in CV risk, HF, and post-MI cohorts, but not in population-based cohorts. However, the CG-BSA discriminative improvement was globally low compared to MDRD4 and especially CKD-EPI formulas. In addition, CG-BSA is inaccurate for renal function estimation and has limited generalizability due to the need for individual height and weight values. Therefore, the CKD-EPI formula offers the best “global package” of renal function estimation and CVM prediction.

## Additional file

Additional file 1: Supplemental Material. **Table S1**. Linearity tests for each formula using restricted cubic splines with 3 knots. **Table S2**. Cox-regression models according to the different formulas (categorized for “renal function” stages) and age subgroups. **Table S3**. Improvement indices for glomerular filtration rate estimation formulas in comparison to body surface area. **Figure S1**. Association between “renal function” formulas and cardiovascular mortality in each population setting using restricted cubic splines with y-axis in log scale. **Figure S2**. Calibration assessment for “renal function” formulas within each population. (DOCX 883 kb)
