# Supplementary material for: Renal function estimation and Cockcroft–Gault formulas for predicting cardiovascular mortality in population-based, cardiovascular risk, heart failure and post-myocardial infarction cohorts: The Heart ‘OMics’ in AGEing (HOMAGE) and the high-risk myocardial infarction database initiatives
Source: BMC Med. 2016 Nov 10;14:181. doi: 10.1186/s12916-016-0731-2 (PMC5103492; doi:10.1186/s12916-016-0731-2)
Supplement: Additional file 1: — Supplemental Material. Table S1. Linearity tests for each formula using restricted cubic splines with 3 knots. Table S2. Cox-regression models according to the different formulas (categorized for “renal function” stages) and age subgroups. Table S3. Improvement indices for glomerular filtration rate estimation formulas in comparison to body surface area. Figure S1. Association between “renal function” formulas and cardiovascular mortality in each population setting using restricted cubic splines with y-axis in log scale. Figure S2. Calibration assessment for “renal function” formulas within each population. (DOCX 883 kb) [file 12916_2016_731_MOESM1_ESM.docx]

**Supplemental Material**

Supplemental Table 1. Linearity tests for each formula using restricted cubic splines with 3 knots.

| **Populations** | **Univariable model** | **Adjusted model*** |
| --- | --- | --- |
| **Population-Based** | | |
| MDRD4 | **0.0007** | **0.006** |
| CKD-EPI | 0.66 | 0.75 |
| CG-BSA | 0.53 | 0.58 |
| **Cardiovascular Risk** | | |
| MDRD4 | **<0.0001** | **<0.0001** |
| CKD-EPI | **<0.0001** | **<0.0001** |
| CG-BSA | **<0.0001** | **<0.0001** |
| **Heart Failure** | | |
| MDRD4 | **0.049** | **0.017** |
| CKD-EPI | 0.57 | 0.32 |
| CG-BSA | 0.23 | 0.095 |
| **Post-Myocardial Infarction** | | |
| MDRD4 | **<0.0001** | **<0.0001** |
| CKD-EPI | **<0.0001** | **<0.0001** |
| CG-BSA | **<0.0001** | **<0.0001** |

Legend: CG-BSA, Cockcroft-Gault formula adjusted for body surface area; MDRD4_,_ modification of diet in renal disease-4 formula; CKD-EPI_,_ Chronic Kidney Disease Epidemiology Collaboration equation.

*Adjusted for gender, smoking status, hypertension history, diagnosis of diabetes, heart rate and systolic blood pressure.

Supplemental Table 2. Cox-regression models according to the different formulas (categorized for “renal function” stages) and age subgroups

| **Variables** | | **HR (95%CI)** | **P-value** | **P for interaction** |
| --- | --- | --- | --- | --- |
| **Community** | | | | |
| **MDRD4** | | | | |
|  | Categorical |  |  | 0.739 |
|  | Stage 1: >=90 | Reference |  |  |
| Age ≤68 | Stage 2: 60 – 89 | 0.778 (0.358 - 1.691) | 0.527 |  |
|  | Stage 3a: 45 – 59 | 1.771 (0.593 - 5.290) | 0.306 |  |
|  | Stage 3b/4/5: <45 | Error (insufficient data) | - |  |
|  | Categorical |  |  |  |
|  | Stage 1: >=90 | Reference |  |  |
| Age >68 | Stage 2: 60 – 89 | 0.732 (0.455 - 1.177) | 0.198 |  |
|  | Stage 3a: 45 – 59 | 0.908 (0.515 - 1.603) | 0.740 |  |
|  | Stage 3b/4/5: <45 | 1.961 (0.992 - 3.874) | 0.053 |  |
| **CKD-EPI** | | | |  |
|  | Categorical |  |  | 0.494 |
|  | Stage 1: >=90 | Reference |  |  |
| Age ≤68 | Stage 2: 60 – 89 | 1.511 (0.603 - 3.783) | 0.378 |  |
|  | Stage 3a: 45 – 59 | 3.501 (0.986 – 12.429) | 0.053 |  |
|  | Stage 3b/4/5: <45 | Error (insufficient data) | - |  |
|  | Categorical |  |  |  |
|  | Stage 1: >=90 | Reference |  |  |
| Age >68 | Stage 2: 60 – 89 | 0.873 (0.403 - 1.893) | 0.731 |  |
|  | Stage 3a: 45 – 59 | 1.145 (0.506 – 2.591) | 0.745 |  |
|  | Stage 3b/4/5: <45 | 2.094 (0.874 – 5.017) | 0.097 |  |
| **CG-BSA** | | | |  |
|  | Categorical |  |  | 0.147 |
|  | Stage 1: >=90 | Reference |  |  |
| Age ≤68 | Stage 2: 60 – 89 | 2.919 (1.002 – 8.507) | 0.050 |  |
|  | Stage 3a: 45 – 59 | 4.664 (1.159 – 18.762) | 0.030 |  |
|  | Stage 3b/4/5: <45 | Error (insufficient data) | - |  |
|  | Categorical |  |  |  |
|  | Stage 1: >=90 | Reference |  |  |
| Age >68 | Stage 2: 60 – 89 | 0.662 (0.300 - 1.459) | 0.306 |  |
|  | Stage 3a: 45 – 59 | 0.925 (0.419 – 2.045) | 0.848 |  |
|  | Stage 3b/4/5: <45 | 1.546 (0.674 – 3.543) | 0.303 |  |
| **Cardiovascular Risk** | | | |  |
| **MDRD4** | | | |  |
|  | Categorical |  |  | <0.001 |
|  | Stage 1: >=90 | Reference |  |  |
| Age ≤70 | Stage 2: 60 – 89 | 0.764 (0.549 - 1.063) | 0.110 |  |
|  | Stage 3a: 45 – 59 | 0.828 (0.565 – 1.214) | 0.334 |  |
|  | Stage 3b/4/5: <45 | 2.518 (1.504 – 4.216) | <0.001 |  |
|  | Categorical |  |  |  |
|  | Stage 1: >=90 | Reference |  |  |
| Age >70 | Stage 2: 60 – 89 | 0.720 (0.516 - 1.004) | 0.052 |  |
|  | Stage 3a: 45 – 59 | 0.792 (0.567 - 1.108) | 0.174 |  |
|  | Stage 3b/4/5: <45 | 1.551 (1.096 – 2.196) | 0.013 |  |
| **CKD-EPI** | | | |  |
|  | Categorical |  |  | <0.001 |
|  | Stage 1: >=90 | Reference |  |  |
| Age ≤70 | Stage 2: 60 – 89 | 0.815 (0.599 - 1.108) | 0.191 |  |
|  | Stage 3a: 45 – 59 | 0.965 (0.675 – 1.378) | 0.843 |  |
|  | Stage 3b/4/5: <45 | 2.920 (1.835 – 4.649) | <0.001 |  |
|  | Categorical |  |  |  |
|  | Stage 1: >=90 | Reference |  |  |
| Age >70 | Stage 2: 60 – 89 | 0.664 (0.330 - 1.336) | 0.251 |  |
|  | Stage 3a: 45 – 59 | 0.699 (0.347 – 1.406) | 0.315 |  |
|  | Stage 3b/4/5: <45 | 1.260 (0.624 – 2.543) | 0.520 |  |
| **CG-BSA** | | | |  |
|  | Categorical |  |  | <0.001 |
|  | Stage 1: >=90 | Reference |  |  |
| Age ≤70 | Stage 2: 60 – 89 | 0.985 (0.760 – 1.277) | 0.907 |  |
|  | Stage 3a: 45 – 59 | 1.489 (1.086 – 2.042) | 0.013 |  |
|  | Stage 3b/4/5: <45 | 3.656 (2.149 – 6.221) | <0.001 |  |
|  | Categorical |  |  |  |
|  | Stage 1: >=90 | Reference |  |  |
| Age >70 | Stage 2: 60 – 89 | 0.627 (0.279 - 1.408) | 0.258 |  |
|  | Stage 3a: 45 – 59 | 0.816 (0.365 – 1.824) | 0.620 |  |
|  | Stage 3b/4/5: <45 | 1.442 (0.644 – 3.230) | 0.373 |  |
| **Heart Failure** | | | | |
| **MDRD4** | | | | |
|  | Categorical |  |  | 0.040 |
|  | Stage 1: >=90 | Reference |  |  |
| Age ≤75 | Stage 2: 60 – 89 | 1.525 (0.818 - 2.843) | 0.184 |  |
|  | Stage 3a: 45 – 59 | 2.039 (1.046 – 3.974) | 0.036 |  |
|  | Stage 3b/4/5: <45 | 3.727 (1.952 – 7.114) | <0.001 |  |
|  | Categorical |  |  |  |
|  | Stage 1: >=90 | Reference |  |  |
| Age >75 | Stage 2: 60 – 89 | 0.584 (0.358 – 0.954) | 0.032 |  |
|  | Stage 3a: 45 – 59 | 0.695 (0.425 - 1.136) | 0.147 |  |
|  | Stage 3b/4/5: <45 | 1.112 (0.694 – 1.782) | 0.659 |  |
| **CKD-EPI** | | | | |
|  | Categorical |  |  | 0.061 |
|  | Stage 1: >=90 | Reference |  |  |
| Age ≤75 | Stage 2: 60 – 89 | 1.362 (0.726 - 2.554) | 0.336 |  |
|  | Stage 3a: 45 – 59 | 2.265 (1.170 – 4.386) | 0.015 |  |
|  | Stage 3b/4/5: <45 | 3.369 (1.780 – 6.373) | <0.001 |  |
|  | Categorical |  |  |  |
|  | Stage 1: >=90 | Reference |  |  |
| Age >75 | Stage 2: 60 – 89 | 1.715 (0.238 – 12.341) | 0.592 |  |
|  | Stage 3a: 45 – 59 | 1.301 (0.180 – 9.376) | 0.794 |  |
|  | Stage 3b/4/5: <45 | 2.706 (0.379 – 19.344) | 0.321 |  |
| **CG-BSA** | | | | |
|  | Categorical |  |  | 0.003 |
|  | Stage 1: >=90 | Reference |  |  |
| Age ≤75 | Stage 2: 60 – 89 | 1.303 (0.668 – 2.543) | 0.437 |  |
|  | Stage 3a: 45 – 59 | 2.967 (1.527 – 5.766) | 0.001 |  |
|  | Stage 3b/4/5: <45 | 5.026 (2.608 – 9.687) | <0.001 |  |
|  | Categorical |  |  |  |
|  | Stage 1: >=90 | Reference |  |  |
| Age >75 | Stage 2: 60 – 89 | 1.499 (0.205 – 10.958) | 0.690 |  |
|  | Stage 3a: 45 – 59 | 1.199 (0.166 – 8.648) | 0.857 |  |
|  | Stage 3b/4/5: <45 | 2.373 (0.332 – 26.943) | 0.389 |  |
| **Myocardial Infarction** | | | | |
| **MDRD4** | | | | |
|  | Categorical |  |  | 0.008 |
|  | Stage 1: >=90 | Reference |  |  |
| Age ≤65 | Stage 2: 60 – 89 | 1.030 (0.895 - 1.185) | 0.681 |  |
|  | Stage 3a: 45 – 59 | 1.863 (1.570 – 2.211) | <0.001 |  |
|  | Stage 3b: 30 - 45 | 2.977 (2.366 – 3.746) | <0.001 |  |
|  | Stage 4/5: <30 | 5.391 (3.256 – 8,926) | <0.001 |  |
|  | Categorical |  |  |  |
|  | Stage 1: >=90 | Reference |  |  |
| Age >65 | Stage 2: 60 – 89 | 1.041 (0.896 – 1.211) | 0.599 |  |
|  | Stage 3a: 45 – 59 | 1.477 (1.269 - 1.720) | <0.001 |  |
|  | Stage 3b: 30 - 45 | 2.314 (1.978 – 2.707) | <0.001 |  |
|  | Stage 4/5: <30 | 3.450 (2.806 – 4.242) | <0.001 |  |
| **CKD-EPI** | | | | |
|  | Categorical |  |  | 0.020 |
|  | Stage 1: >=90 | Reference |  |  |
| Age ≤65 | Stage 2: 60 – 89 | 1.097 (0.956 - 1.258) | 0.336 |  |
|  | Stage 3a: 45 – 59 | 1.924 (1.624 – 2.279) | <0.001 |  |
|  | Stage 3b: 30 - 45 | 3.173 (2.532 – 3.977) | <0.001 |  |
|  | Stage 4/5: <30 | 4.629 (2.799 – 7.655) | <0.001 |  |
|  | Categorical |  |  |  |
|  | Stage 1: >=90 | Reference |  |  |
| Age >65 | Stage 2: 60 – 89 | 1.103 (0.896 – 1.358) | 0.356 |  |
|  | Stage 3a: 45 – 59 | 1.538 (1.250 – 1.894) | <0.001 |  |
|  | Stage 3b: 30 - 45 | 2.404 (1.949 – 2.966) | <0.001 |  |
|  | Stage 4/5: <30 | 3.671 (2.911 – 4.629) | <0.001 |  |
| **CG-BSA** | | | | |
|  | Categorical |  |  | <0.001 |
|  | Stage 1: >=90 | Reference |  |  |
| Age ≤65 | Stage 2: 60 – 89 | 1.209 (1.056 – 1.385) | 0.006 |  |
|  | Stage 3a: 45 – 59 | 2.436 (2.052 – 2.893) | <0.001 |  |
|  | Stage 3b: 30 - 45 | 3.549 (2.719 – 4.632) | <0.001 |  |
|  | Stage 4/5: <30 | 11.311 (5.346 – 23.933) | <0.001 |  |
|  | Categorical |  |  |  |
|  | Stage 1: >=90 | Reference |  |  |
| Age >65 | Stage 2: 60 – 89 | 0.989 (0.773 – 1.266) | 0.930 |  |
|  | Stage 3a: 45 – 59 | 1.396 (1.095 – 1.779) | 0.007 |  |
|  | Stage 3b/4/5: <45 | 2.404 (1.886 – 3.064) | <0.001 |  |
|  | Stage 4/5: <30 | 3.685 (2.841 – 4.780) | <0.001 |  |

Legend: all analyses are univariable.

CG-BSA, Cockcroft-Gault formula adjusted for body surface area; MDRD4_,_ modification of diet in renal disease-4 formula; CKD-EPI_,_ Chronic Kidney Disease Epidemiology Collaboration equation.

Supplemental Table 3. Improvement indices for glomerular filtration rate estimation formulas in comparison to body surface area.

| **Improvement indices**  **and populations** | **MDRD4 on top of BSA** | **P-value** | **CKD-EPI on top of BSA** | **P-value** | **CG-BSA on top of BSA** | **P-value** |
| --- | --- | --- | --- | --- | --- | --- |
| **Community** | | | | | | |
| **NRI continuous**  **2 yr (95%CI)** | 0.096 (-0.019 to 0.257) | 0.132 | 0.195 (0.030 to 0.299) | 0.013 | 0.240 (0.097 to 0.361) | <0.001 |
| **IDI continuous**  **2 yr (_95%_CI)** | 0.002 (0.000 to 0.008) | 0.006 | 0.006 (0.002 to 0.013) | <0.001 | 0.010 (0.004 to 0.019) | <0.001 |
| **Cardiovascular Risk** | | | | | | |
| **NRI continuous**  **2 yr (95%CI)** | 0.079 (0.040 to 0.118) | <0.001 | 0.111 (0.080 to 0.147) | <0.001 | 0.208 (0.171 to 0.238) | <0.001 |
| **IDI continuous**  **2 yr (_95%_CI)** | 0.003 (0.002 to 0.006) | <0.001 | 0.006 (0.004 to 0.010) | <0.001 | 0.012 (0.009 to 0.017) | <0.001 |
| **Heart Failure** | | | | | | |
| **NRI continuous**  **2 yr (95%CI)** | 0.192 (0.112 to 0.260) | <0.001 | 0.205 (0.120 to 0.275) | <0.001 | 0.256 (0.188 to 0.315) | <0.001 |
| **IDI continuous**  **2 yr (_95%_CI)** | 0.027 (0.014 to 0.044) | <0.001 | 0.034 (0.019 to 0.047) | <0.001 | 0.042 (0.027 to 0.058) | <0.001 |
| **Improvement indices**  **and populations** | **BSA on top of MDRD4** | **P-value** | **BSA on top of CKD-EPI** | **P-value** | **BSA on top of CG-BSA** | **P-value** |
| **Community** |  |  |  |  |  |  |
| **NRI continuous**  **2 yr (95%CI)** | -0.017 (-0.133 to 0.176) | 1.000 | -0.001 (-0.138 to 0.129) | 0.977 | 0.075 (-0.055 to 0.197) | 0.266 |
| **IDI continuous**  **2 yr (_95%_CI)** | -0.000 (-0.000 to 0.000) | 1.000 | 0.000 (0.000 to 0.000) | 1.000 | 0.001 (-0.000 to 0.000) | 0.285 |
| **Cardiovascular Risk** |  |  |  |  |  |  |
| **NRI continuous**  **2 yr (95%CI)** | 0.101 (0.058 to 0.118) | <0.001 | 0.093 (0.051 to 0.131) | <0.001 | -0.006 (-0.041 to 0.051) | 1.000 |
| **IDI continuous**  **2 yr (_95%_CI)** | 0.002 (0.001 to 0.006) | <0.001 | 0.002 (0.001 to 0.003) | <0.001 | 0.000 (0.000 to 0.000) | 1.000 |
| **Heart Failure** |  |  |  |  |  |  |
| **NRI continuous**  **2 yr (95%CI)** | 0.132 (0.041 to 0.201) | <0.001 | 0.124 (0.034 to 0.201) | <0.001 | 0.065 (-0.037 to 0.043) | 0.229 |
| **IDI continuous**  **2 yr (_95%_CI)** | 0.013 (0.004 to 0.024) | <0.001 | 0.012 (0.004 to 0.021) | <0.001 | 0.003 (0.000 to 0.009) | 0.053 |

Legend: CG-BSA, Cockcroft-Gault formula adjusted for body surface area; MDRD4_,_ modification of diet in renal disease-4 formula; CKD-EPI_,_ Chronic Kidney Disease Epidemiology Collaboration equation; BSA, body surface area.

Supplemental Figure 1. Association between “renal function” formulas and cardiovascular mortality in each population setting using restricted cubic splines with y-axis in log scale.


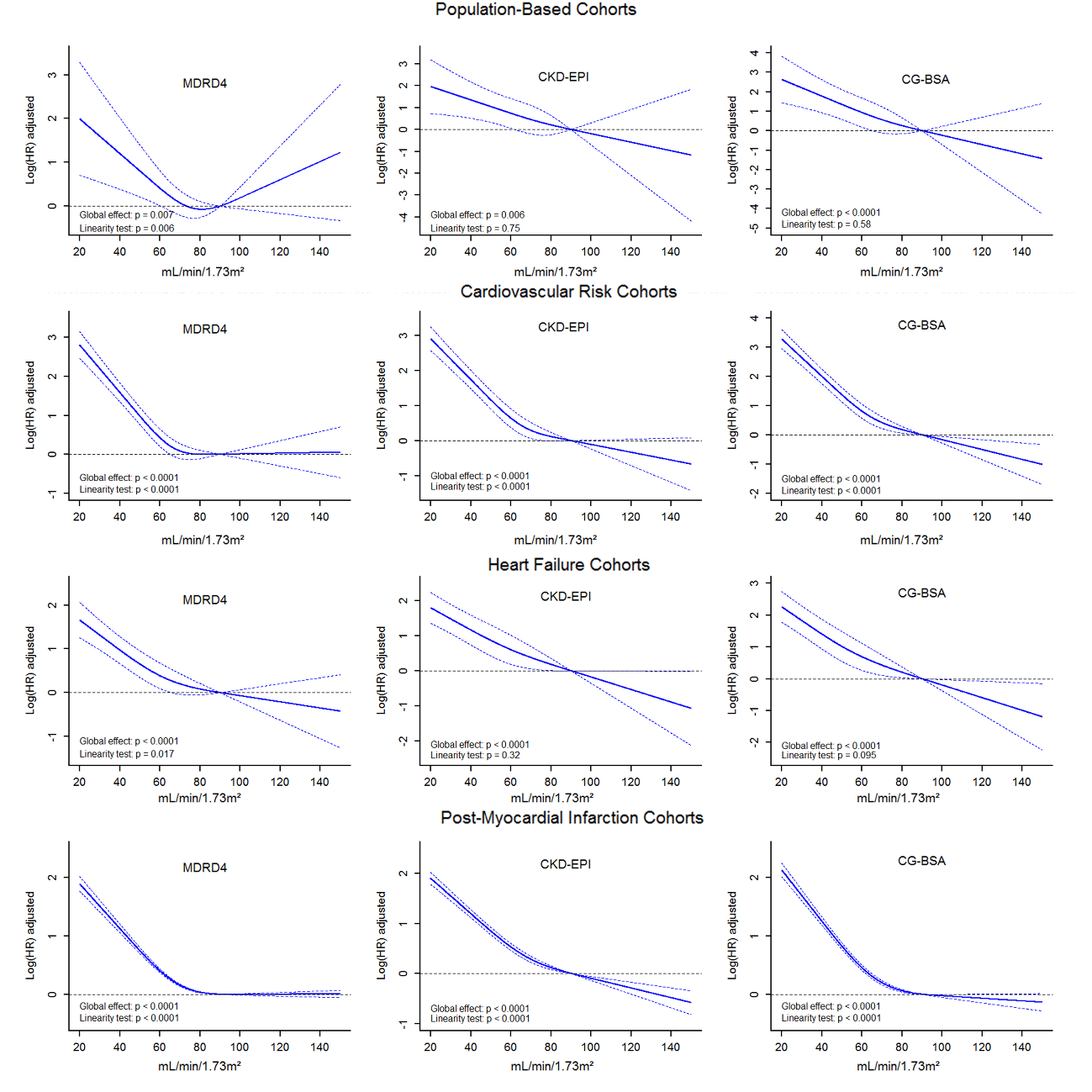


Legend: CG-BSA, Cockcroft-Gault formula adjusted for body surface area; MDRD4_,_ modification of diet in renal disease-4 formula; CKD-EPI_,_ Chronic Kidney Disease Epidemiology Collaboration equation.

Models adjusted for gender, smoking status, hypertension history, diagnosis of diabetes, heart rate and systolic blood pressure.

Supplemental Figure 2. Calibration assessment for “renal function” formulas within each population


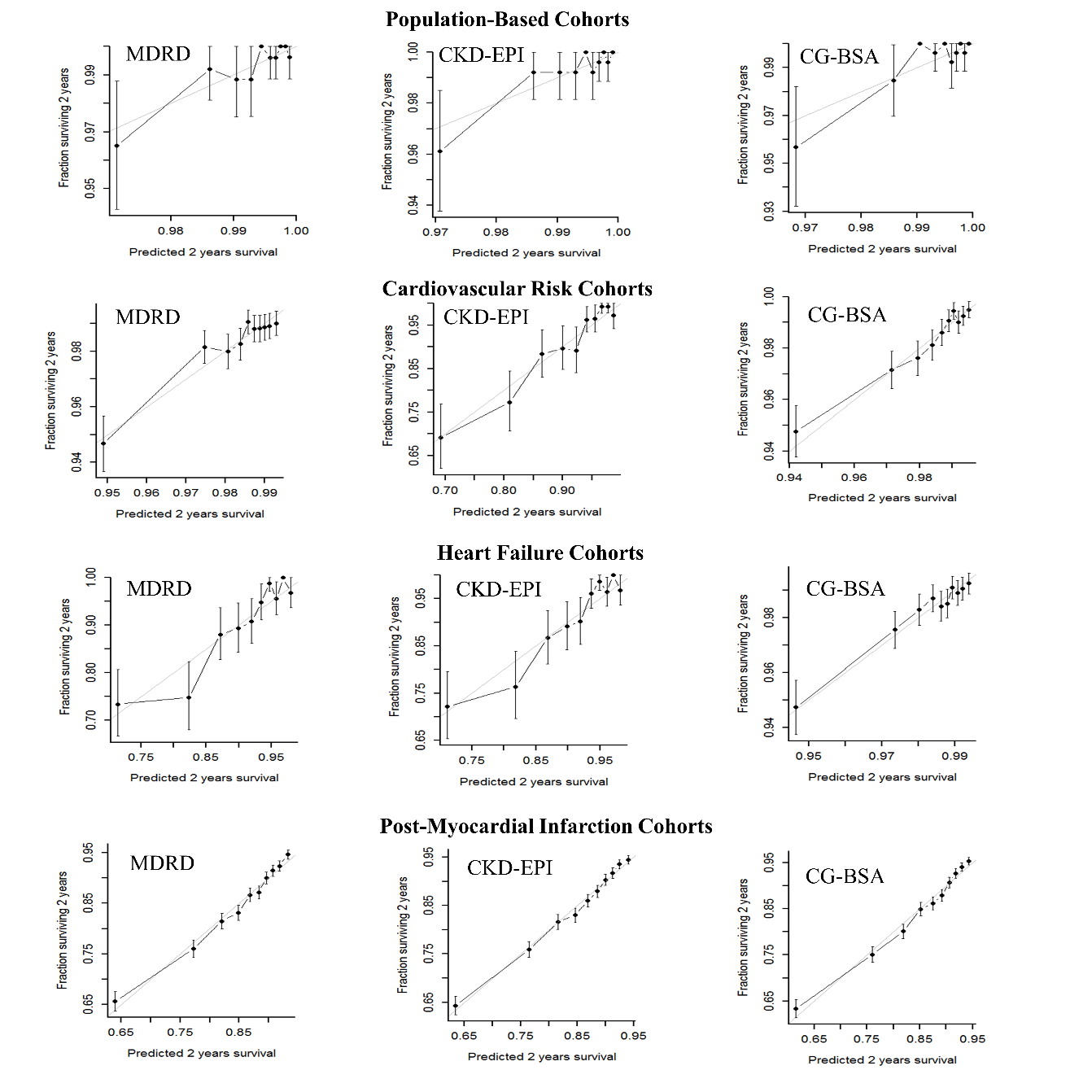


Legend: CG-BSA, Cockcroft-Gault formula adjusted for body surface area; MDRD4_,_ modification of diet in renal disease-4 formula; CKD-EPI_,_ Chronic Kidney Disease Epidemiology Collaboration equation.

The outcome is cardiovascular mortality.

Dots represent the observed survival among deciles of predicted risks whereas the thin line represents the predicted risk values derived from Cox models.
